# The Gut Microbiome and Metastatic Renal Cell Carcinoma

**DOI:** 10.3390/jcm12041502

**Published:** 2023-02-14

**Authors:** Luis Meza, Matthew Feng, Kyle Lee, Rubens Sperandio, Sumanta Kumar Pal

**Affiliations:** 1 Department of Medical Oncology & Experimental Therapeutics, City of Hope Comprehensive Cancer Center, Duarte, CA 91010, USA; 2 Hospital Israelita Albert Einstein, São Paulo 05652-900, Brazil

**Keywords:** renal cell carcinoma, gut microbiome, translational research

## Abstract

The introduction of targeted therapy (TT) and immuno-oncology (IO) agents have revolutionized the treatment of metastatic renal cell carcinoma (mRCC). However, despite the significant improvements in survival and clinical response yielded by these agents, a significant percentage of patients still experience progressive disease. Evidence now suggests that microorganisms living in the gut (i.e., the gut microbiome) could be used as a biomarker for response and may also have utility in increasing response to these treatments. In this review, we present an overview of the role of the gut microbiome in cancer and its potential implications in the treatment of mRCC.

## 1. Introduction

Approximately 82,000 new cases of kidney cancer will be diagnosed in the United States during 2023, with varying rates of progression to metastatic disease [1]. While treatment options for localized disease have remained largely unchanged, significant advances have occurred in the treatment landscape of metastatic renal cell carcinoma (mRCC). The last couple of decades have seen an explosion in the number of U.S. Food and Drug Administration (FDA) approvals for this disease setting with multiple targeted-therapy agents (TT), and immune checkpoint inhibitors (ICIs) being now available for this patient population. TT agents can be divided into (a) inhibitors of vascular endothelial growth factor (VEGF) signaling, which include drugs such as sunitinib, pazopanib, axitinib, cabozantinib and levantinib, and (b) inhibitors of the mammalian target of rapamycin (mTOR), represented by everolimus and temsirolimus [2,3,4,5,6,7]. In contrast, ICIs block coinhibitory molecules such as programmed death-1 (PD-1), the programmed death ligand-1 (PD-L1) and the cytotoxic T-lymphocyte activating protein-4 (CTLA-4) [8].

Despite the numerous available options for patients with mRCC with the use of the previously mentioned agents, either as monotherapy or in combination, response to these regimens remains heterogeneous, with some patients achieving a complete response (CR) while others experience progressive disease (PD). Moreover, the 5-year survival rate for patients in this stage is only 15% [9]. Therefore, selecting the approach that will yield the most benefit for a given patient remains a significant challenge [10]. Despite multiple efforts to identify biomarkers predictive of response, such as the gene expression signatures from the IMmotion 151 trial, tumor mutational burden (TMB), and PD-L1 expression, the International mRCC Database Consortium (IMDC) risk model remains the only predictive biomarker to be prospectively validated in a phase 3 trial to date. There is, therefore, a need to increase our understanding of the biological processes underlying the development and evolution of RCC to develop novel biomarkers of response that will allow for treatment selection in an individualized manner.

In recent years, fueled by the advent of next-generation sequencing technologies, there has been an increased interest in the evaluation of the gut microbiome and its role in cancer. Multiple studies now show that certain bacterial species might be associated with the development of certain cancers such as lung, melanoma and colon, as well as with treatment response to currently available regimens [11]. In the setting of RCC, there have also been efforts to characterize the role of the gut microbiome. Here, we provide an overview of the role of the gut microbiome in cancer with a special focus on RCC. In addition, we highlight the ongoing trials in the field and discuss the importance of intra–inter-institutional collaboration for creating a solid working framework for microbiome studies in the future.

## 2. Gut Microbiome

It is estimated that the human body is composed of around 3.7 × 1013 human cells [12]. In addition to these cells, the healthy human body also comprises a plethora of microbes including bacteria, viruses and fungi which are collectively known as the microbiome. Revised estimates suggest that these organisms amount to at least 3.8 × 1013 cells, accounting for approximately half of the total number of cells present in the body, and are intrinsically involved in the regulation and maintenance of human health [13,14]. However, although these organisms can be found in multiple tissues throughout the human body, such as in the skin, oral mucosa, and gastrointestinal tract, it is this last one, particularly the colon, that hosts the highest number of bacteria, exceeding all other organs by two orders of magnitude [13]. It is well established that the gut microbiome plays an integral role in a number of physiologic functions that include the metabolism and uptake of nutrients, the preservation of the intestinal barrier, and modulation of the immune system [14]. Indeed, it is now known that there is a complex interplay between the gut microbiota and the immune system of the host that impacts both local immunity and peripheral white blood cell dynamics [15,16,17,18,19,20,21,22].

It has been hypothesized that intestinal microbes confer many metabolic capabilities needed for the preservation of the host’s immune homeostasis and that alterations of the gut microbiome composition (dysbiosis) could lead to immune alterations contributing to the development of a number of systemic disorders [23,24] Notably, numerous studies have shown its association with a number of inflammatory and autoimmune conditions such as inflammatory bowel disease and lupus nephritis, while a number of persuasive interventional studies have further demonstrated that microbiome modulating strategies, such as fecal microbiota transplantation (FMT), can induce remission of some of these conditions and modulate treatment response [25,26,27,28,29].

## 3. Gut Microbiome and Cancer

It is therefore not surprising that given the successes in establishing associations between the gut microbiome and several diseases, subsequent studies have sought to determine its influence in the context of cancer. Interestingly, and despite the increased interest in examining the role of the microbiome in cancer seen in recent years, there are historical reports dating back to 1868 suggesting a link between the presence of certain microbes and oncogenesis [11,30,31]. Among the microbes reported to have a role in carcinogenesis are viruses such as the Epstein–Barr, human papilloma, and hepatitis viruses and bacteria such as *Helicobacter pylori* [32,33]. Nevertheless, the path to characterization of other microbiome–cancer associations has been largely truncated by technical challenges of the time. Encouragingly, the advent of new laboratory techniques and technologies such as next-generation genomic sequencing is helping us to deepen our understanding of the contribution of bacteria present in the gut to the development of cancer and their influence in response to anti-cancer systemic therapies and their associated toxicities [34].

It is through the incorporation of these new technologies that pivotal investigations have been able to show the presence of distinct microbial profiles in the gut of cancer patients compared with their cancer-free counterparts [34,35]. Moreover, the preponderance of preclinical and clinical evidence now suggests that gut dysbiosis plays key role in the natural history of a number of malignancies including colorectal cancer, hepatocellular carcinoma, melanoma and breast cancer [36,37,38,39,40,41]. Furthermore, the influence of the gut microbiome has been investigated in the setting of different systemic therapy approaches, such as chemotherapy, stem cell transplantation and immunotherapy, where it has been shown to modulate toxicity and treatment response [31,42,43,44,45,46]. Particularly, significant efforts have been dedicated to investigating the association between the gut microbiome and immune-related adverse events (irAEs). Evidence now suggests that differences in gut microbiome profiles exist between patients who experience irAEs and those who do not [47,48,49]. This finding could potentially be used to develop biomarkers to predict their occurrence prior to initiation of therapy, as well as devising interventions to abrogate these events once they ensue [49].

Notably, associations between certain bacterial species and response to immune checkpoint blockade (anti-CTLA-4 and anti-PD-1) have also been demonstrated across different cancer types, suggesting the presence of “responder” and “non-responder” gut microbiome profiles [50,51,52,53] Indeed, there have been several efforts to recapitulate these favorable profiles through interventions such as FMT or bacterial supplementation that have shown some success in enhancing therapeutic response and overcoming resistance [50,54,55,56,57]. Likewise, dietary changes such as a higher fiber intake have also been associated with an increased benefit from ICIs in preclinical and clinical models [58]. All of the compounding evidence has resulted in the inclusion of “polymorphic microbes” as a new emerging hallmark of cancer [59,60]. However, despite these encouraging data, the cellular and molecular underpinnings that critically regulate these interactions are yet to be completely elucidated.

Although not fully understood, it is thought that the gut microbiome influences host immunity and carcinogenesis through positive and negative interaction with other recognized hallmarks of cancer [59]. This is mediated by a number of mechanisms including (1) direct DNA damage and the disruption of systems that aim to maintain genomic integrity, (2) production of ligand mimetics that stimulate epithelial proliferation, (3) secretion of gut hormones, (4) elicitation of immune responses through cross-reactive microbial and tumor-associated antigens and (5) shifts in the gut ecosystem causing changes in the levels of microbial metabolites [34,61,62,63,64,65,66]. Whereas it is certainly challenging to ascertain which of these factors has the biggest influence in the context of cancer, there is an increasing body of evidence suggesting that microbial metabolites and secondary metabolites not only play a key role in the onset and development of numerous malignancies, but could also be drivers of response of systemic treatment, namely immunotherapy [48,50,51,54,67]. One such group of metabolites are short-chain fatty acids (SCFAs), such as butyrate and propionate, which originate from the bacterial fermentation of non-digestible carbohydrates, and have been implicated in the reduction of inflammation and regulation of CD4+ and CD8+ T cells [66,68,69,70,71,72,73]. Moreover, butyrate has also been shown to have a role in tumor suppression through the up- and down-regulation of genes involved in carcinogenesis [66,74,75]. Indeed, this SCFA seems to induce a pro-apoptotic effect through the increased expression of genes such as *Bax* and *Bak*, and has been proposed to have an additional tumor suppressing effect by regulating the *Wnt/*β-catenin signaling pathway and by reducing the expression of anti-apoptotic genes such as *Bcl-2* [73,74,76,77,78].

## 4. Gut Microbiome and Renal Cell Carcinoma

The treatment of metastatic renal cell carcinoma has changed dramatically over the past decades with the introduction of targeted treatment strategies with tyrosine kinase inhibitors such as sunitinib, pazopanib and cabozantinib and more recently with the approval of ICIs that target inhibitory molecules such as PD-1, PD-L1 and CTDLA-4 [8]. The use of this latter treatment modality, either alone or in combination with TT, has further improved the outcome of patients with mRCC and is currently the standard of care for first-line treatment of this disease. However, unlike other malignancies such as non-small cell lung cancer and melanoma, where the use of ICIs can be guided by PD-L1 tumor expression or tumor mutational burden, there are currently no validated biomarkers to predict response in patients with mRCC receiving ICIs [79,80,81,82]. Moreover, despite the improvements in efficacy seen with current treatment approaches, up to 60% of patients receiving these regimens fail to respond [83]. Hence, there is increasing need for both biomarkers of response that will allow us to identify the group of patients that will benefit the most from these treatments, and interventions that can allow us to maximize the benefit conferred by these approaches.

Given this context, as well as the large body of evidence linking the gut microbiome with the host’s immune system and treatment response to ICIs in other malignancies, the role of the gut microbiome in mRCC and its potential as a biomarker of response and an intervention to improve treatment effectiveness are also being studied. Initial observations from several studies, the majority of which were retrospective in nature, have sought to indirectly determine the impact of gut dysbiosis in treatment response to ICIs by assessing for changes in the context of antibiotic treatment. Overall, the resulting evidence indicates that treatment with antibiotics is associated with decreased overall survival (OS), progression-free survival (PFS) and objective response rate (ORR) in patients with mRCC treated with standard-of-care ICIs [84]. Moreover, a study by De Rosa and colleagues further suggested that antibiotic treatment was associated with an alteration in the composition of the intestinal microbiota and the taxonomic beta diversity. Namely, this study noted an over-representation of bacteria, such as *Erysipelotrichaceae bacterium* and *Clostridium hathewayi*, suggesting that akin to the observations made for other cancer types, gut dysbiosis could also affect treatment response in RCC [85].

Additional studies further extended this line of inquiry and aimed to delineate this effect by assessing the impact of baseline gut microbiome profiles in patients receiving ICIs. This was performed by collecting stool specimens prior to the initiation of treatment and looking for the relative abundance of different bacteria using whole genome sequencing (WGS). These studies found that an increase in microbial diversity, as well as in relative abundance of certain bacterial species such as *Akkermansia muciniphila* and *Bifidobacterium spp.*, was associated with response to ICIs [50,85,86]. In contrast, data published by Park and colleagues who evaluated a cohort of NSCLC and RCC patients showed that a lack of treatment response was associated with an over-representation of the *Enterocloster* genus [85]. Further work presented by Alves during the 2022 ESMO symposium supported these findings, noting that not only was the baseline overrepresentation of the *Enterocloster* genus linked with a lack of treatment response but that those patients who do respond to ICIs exhibited a decrease in the *Enterocloster* genus representation after treatment [87].

Preclinical models have been in turn devised to evaluate the impact of gut microbiome interventions in treatment response and have shown that the direct administration of bacterial species associated with response in previous studies, such as *Bifidobacterium* and *Akkermancia muciniphila*, could delay tumor progression and restore treatment efficacy in mice treated with an immune checkpoint blockade [50,88]. Interestingly, it has also been shown that bacterial supplementation with *Clostridium butyricum MIYAIRI 588 (CBM 588)*, a probiotic bacterium, could lead to an increase in relative abundance of previously identified “beneficial bacteria” such as *Bifidobacterium* and *Lactobacillus* in mice, while also enhancing the intestinal barrier function [89].

Current studies in humans have intended to harness this effect to achieve an increased response to treatment and a reduction in treatment-related side effects using several strategies including (1) bacterial supplementation, (2) fecal microbiota transplantation and (3) diet modulation.

The first randomized clinical trial in this space was conducted by Dizman and colleagues. In the study, twenty patients with mRCC who were initiating VEGF-TKIs in any line of therapy were randomized to a probiotic-supplemented arm receiving a Bifidobacterium-containing yogurt, or a probiotic-restricted arm. Notably, all patients enrolled to the intervention arm reached detectable levels of *Bifidobacterium animalis*. Although no difference in clinical benefit was seen between these arms, whole metagenome sequencing identified that *Barnesiella intestinihominis* and *Akkermansia muciniphila* were significantly more abundant in patients achieving clinical benefit [90].

Another study was then carried out by the same group evaluating the effect of live bacterial supplementation with CBM588 in treatment naïve mRCC patients receiving ipilimumab with nivolumab for first-line treatment [91]. A total of 30 patients were randomized in a 2:1 fashion to the probiotic-containing and probiotic restricted arms, respectively. Despite the robust preclinical and clinical rationale behind its’ primary endpoint of characterizing the effect of CBM588 on the relative abundance of *Bifidobacterium* spp., this endpoint was not met [54,91]. However, a significant advantage in PFS was seen in those receiving live bacterial supplementation over those receiving ipilimumab with nivolumab alone (12.7 vs. 2.5 months, hazard ratio 0.15, 95% CI 0.05–0.47, *p* < 0.001). Additionally, a comparable safety profile was seen among the two groups, with grade 3 and 4 adverse events being reported in 50% and 52% of patients in the control and intervention arms, respectively.

**Figure 1:**
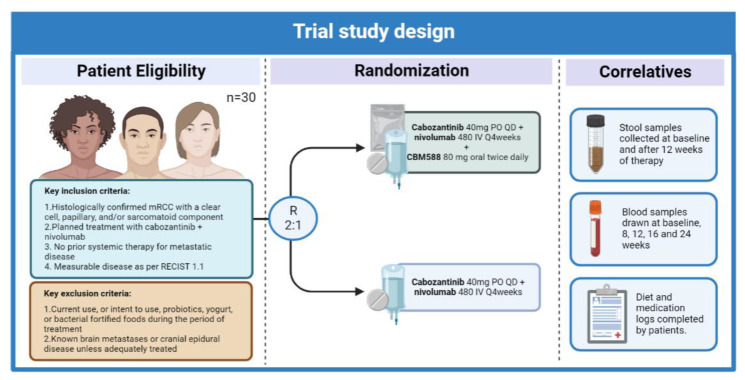
Study design for the phase I cabozantinib plus nivolumab +/− CBM588 trial [97].

Despite this encouraging PFS signal, a remaining question is whether the effects of CBM588 will also be relevant in the context of newer combination strategies combining ICIs and TKIs. This is especially true in light of our growing understanding of the effect of TKIs in immune responses with several pieces of evidence suggesting that common TKI-driven effects such as VEGF blockage or more specific activity such as inhibition of MET and the TAM kinases, as seen with cabozantinib, could play an immunomodulatory role [92,93,94,95]. To answer this question, and given the encouraging safety profile seen in the aforementioned trial, a currently ongoing study will evaluate the effect of CBM588 in treatment-naïve patients receiving treatment with a combination of cabozantinib plus nivolumab as first-line therapy for mRCC [96] (Figure 1).

FMT represents another microbiome-directed intervention with increasing momentum in the treatment of mRCC. Although there are still limited published data regarding the effect of this approach in this disease, current evidence suggests that FMT could improve mucosa-associated invariant T (MAIT) cell function in this patient population and boost immune surveillance against opportunistic pathogens that might be of relevance in the setting of cancer-mediated immunosuppression [98]. Furthermore, this intervention is also being evaluated as a way to reduce treatment-related toxicity. In a study conducted by Ianiro et al., FMT was employed to reduce TKI-induced diarrhea in mRCC patients. In his study, patients treated with donor-FMT showed a significant clinical improvement in TKI-induced diarrhea symptoms when compared to those receiving placebo [99]. Another interesting study is the currently ongoing PERFORM trial, one that will evaluate the prevention of treatment toxicity with immunotherapy utilizing this approach [100].

Beyond this, FMT is being evaluated as a tool to improve and induce response to ICIs in the TACITO and MITRIC trials, respectively. The TACITO trial is a randomized control trial of 50 mRCC patients to receive FMT or placebo and will evaluate the number of participants free of tumor progression [101]. In contrast, the MITRIC trial will enroll patients with solid tumors (including RCC) that have failed to respond to treatment. This is a single-arm, open-label study that will enroll 20 patients who will receive FMT from ICI-responders after experiencing progressive disease while on therapy with PD1/PD-L1 blockers and/or CTLA4-blockers [102]. The rationale behind these trials derives from pre-clinical evidence showing that FMT from patients responding to ICIs can successfully rescue primary resistance in RCC tumor-bearing mice [85]. Moreover, similar concepts have already been successfully implemented in cohorts of immunotherapy-refractory patients with melanoma [56,57].

**Figure 2:**
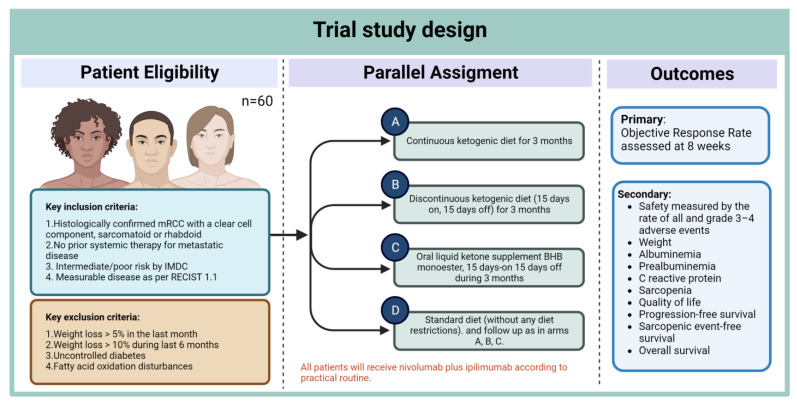
Study design for the KETOREIN trial.

Finally, dietary interventions are also underway in the KETOREIN trial. This is a non-randomized four-arm design that aims to evaluate a ketogenic diet used concomitantly with nivolumab plus ipilimumab in mRCC patients. This trial will evaluate objective response rate as its primary outcome and will enroll a total of 60 patients to one of four arms detailed in Figure 2 [103]. Results from this trial will build upon previously published pre-clinical data from Ferrere et al. suggesting that a ketogenic diet shifts the balance of the gut microbiota from tolerogenic to immunogenic bacteria (e.g., *Akkermancia muciniphila*) and induces an antineoplastic effect mediated by 3-hydroxybutyrate [104].

## 5. Challenges and Opportunities

Historically, challenges related to the characterization of the microbiome were mostly attributable to technical limitations, especially considering that not all regular bacterial species are amenable to culture processes, and that cultivating viruses and fungi can be even more challenging. Moreover, body environments other than the gut are less colonized and have yielded disappointing results. It was only more recently, with the advent of advanced molecular techniques such as DNA sequencing and fluorescence in-situ hybridization of stool, blood and saliva samples, as well as intra-tumoral analysis, that a broader characterization of the human microbiome became independent from culture methods [105,106]. The most utilized tool as a strategy to surpass the challenge of obtaining reliable and high-quality samples is sequencing the 16S rRNA gene, which is present only in prokaryotic cells, with the drawback of identifying only bacteria [106]. Notwithstanding, even when high-throughput sequencing technologies are increasingly available, up to 50% of functional diversity remains unknown, a fact that is further complicated when including non-reference populations [105].

Furthermore, although evolutionary advances in next-generation sequencing technology have ushered in a new understanding of the interplay between the gut microbiome, immunity and cancer, several challenges are notable and represent barriers for its incorporation in routine clinical practice. Among these challenges are the lack of uniformity across the methodologies used for microbiome analysis (e.g., stool collection kits, probiotic restriction in the control arms, etc.), an issue that could explain the modest overlap in gut-microbiome profiles associated with response across studies (Table 1). Hence, the development and validation of a reference framework would be a promising approach to be incorporated in microbiome research that could facilitate collaboration and the comparison of results.

Another important challenge is the limited sample size of most studies. Considering that microbiome profiling can be influenced by factors such as age, diet, socioeconomic status, geography and ethnicity, large sets of data are needed to identify and fully capture this heterogeneity [107]. Joint efforts analogous to The Cancer Genome Atlas (TCGA) could prove beneficial in better understanding the immune–microbiome interface. Initiated in 2006, TCGA consisted of a collaboration across multiple institutions and with the labor of a myriad of multidisciplinary specialists to collect and analyze data from over 20,000 samples across 33 different cancer types to elucidate genomic aspects of cancer. A similar approach would be an important step in microbiome research, with the collection of information from multiple centers, including academic and community sites, able to create a more robust database and provide the foundation for insights into different microbiome compositions. Ongoing population-wide initiatives such as The Human Microbiome Project (HMP) in the United States, the Metagenomes of the Human Intestinal Tract (MetaHIT) in Europe, and a diabetes cohort in China have already managed to survey around 2000 individuals [108,109,110].

The City of Hope is one medical group primed to help in this collaborative effort. With over 30 different locations across Southern California, this network is well positioned to conduct studies that collect samples representative of a broad population. Not only accounting for the diverse ethnic backgrounds present in the state of California, but also socioeconomic and cultural factors, can help broaden the resident microbiota. Additionally, the institution recently broadened its area of influence and cancer care beyond its original regional borders by acquiring the Cancer Treatment Centers of America group, which has a well-established presence in Georgia, Illinois and Arizona. This will hopefully allow for nation-wide studies that will provide a wider look at the composition of what constitutes a normal microbiome and will help better determine the changes seen during treatment and survey differences across various patient groups.

Admittedly, this collaborative endeavor would require the contribution of experts in many areas of biomedical research. Physicians and patient care personnel would identify eligible candidates to provide samples. Basic science researchers would identify strains, elucidate molecular pathways, and understand the gut microbiome’s modulatory effects. In turn, bioinformaticians and data scientists would play a role in identifying correlations and scrutinizing data. With recent studies relating the gut microbiome to cancer treatment response and toxicity, it is particularly important for basic scientists to use animal models to understand the mechanism behind these findings. Communication across all levels of the chain of care is required to streamline such an effort and translate findings to patient care and the clinical setting.

With broad patient samples, physicians from multiple sites, and basic science labs working together, we can broaden our understanding of the mechanisms driving microbiome modulatory effects and use this knowledge to provide more personalized treatment options for patients. For example, in a certain cancer population, if malnutrition or a poor microbiota diversity is identified, we might be able to correct the course of treatment and increase the odds of response and perhaps extend survival by administering live bacterial products, as early-phase data have suggested, with larger confirmatory trials underway [111].

## 6. Conclusions and Future Directions

In summary, the gut microbiome represents an area of emerging interest in oncology. Difficulties faced during initial efforts for the characterization of the vast array of microorganisms that reside in the human body have now been largely addressed by the introduction and use of next-generation sequencing technologies. It is now well accepted that the microorganisms living in the gut have an impact across many disease settings, including cancer, and studies have further implicated the gut microbiome as a potential biomarker for response in many cancer types including mRCC.

Furthermore, randomized clinical trials in the mRCC space have produced encouraging results supporting the use of microbiome-based interventions to increase the effectiveness of systemic therapy and reduce toxicity. Ongoing clinical trials are seeking to validate these findings in larger cohorts, as well as address other clinically relevant questions, including the effect of dietary interventions in treatment outcomes. However, much work remains to be done before microbiome-based interventions can have a tangible impact in routine clinical practice.

Namely, there is an unmet need for longitudinal microbiome data at the individual and population level that can provide insights into the heterogeneity of the gut microbiome across different patient populations. Hence, future research efforts should aim to include diverse patient populations, as well as carefully annotated correlatives including genomic, epigenomic and metabolomic data, all of which will help us elucidate the factors driving differences between patient cohorts. Admittedly, such projects will necessitate large intra- and inter-institutional collaborations which remain, to date, a largely unfulfilled opportunity.

## Figures and Tables

**Table 1:** Studies evaluating gut microbiome composition and treatment response in mRCC patients.

Study	Patient Population	Microorganism Associated with Response/Clinical Benefit	Treatment
Routy et al, Science (2018) [50]	Patients with metastatic RCC or NSCLC	*Akkermansia muciniphila*, *Ruminococcus*, *Alistipes*, and *Eubacterium*	Anti PD-L1
Derosa et al, European Urology, (2020) [85]	Patients with advanced RCC	*Akkermansia muciniphila*, *Bacteroides salyersiae*, and *Eubacterium siraeum*	Nivolumab
Salgia European Urology (2020) [86]	Patients with metastatic RCC	*Akkermansia municiphila*, *Prevotella copri*, *Feacalibacterium rumino Bifidobacterium adolescentis*, and *Barnesiella intestinihominis*	Nivolumab or nivolumab with ipilimumab
Dizman et al, Cancer Medicine (2021) [90]	Patients with metastatic RCC	*Akkermansia muciniphila*, *Barnesiella intestinihominis and Bacteroides caccae*	VEGF-TKI therapy
Dizman et al, Nature Medicine (2022) [91]	Patients with metastatic RCC	*Bifidobacterium* spp.	Nivolumab with ipilimumab +/− CBM588
